# Harnessing RIG-I and intrinsic immunity in the tumor microenvironment for therapeutic cancer treatment

**DOI:** 10.18632/oncotarget.25626

**Published:** 2018-06-22

**Authors:** David L. Elion, Rebecca S. Cook

**Affiliations:** ^1^ Cancer Biology Program, Vanderbilt University School of Medicine, Nashville, TN 37232, USA; ^2^ Department of Cell and Developmental Biology, Vanderbilt University School of Medicine, Nashville, TN 37232, USA; ^3^ Department of Biomedical Engineering, Vanderbilt University School of Engineering, Nashville, TN 37232, USA; ^4^ Vanderbilt Ingram Cancer Center, Vanderbilt University Medical Center, Nashville, TN 37232, USA

**Keywords:** immunotherapy, RIG-I, innate immunity, pyroptosis, tumor microenvironment

## Abstract

Cancer immunotherapies that remove checkpoint restraints on adaptive immunity are gaining clinical momentum. Approaches aimed at intrinsic cellular immunity in the tumor microenvironment are less understood, but are of intense interest, based on their ability to induce tumor cell apoptosis while orchestrating innate and adaptive immune responses against tumor antigens. The intrinsic immune response is initiated by ancient, highly conserved intracellular proteins that detect viral infection. For example, the RIG-I-like receptors (RLRs), a family of related RNA helicases, detect viral oligonucleotide patterns of certain RNA viruses. RLR activation induces immunogenic cell death of virally infected cells, accompanied by increased inflammatory cytokine production, antigen presentation, and antigen-directed immunity against virus antigens. Approaches aimed at non-infectious RIG-I activation in cancers are being tested as a treatment option, with the goal of inducing immunogenic tumor cell death, stimulating production of pro-inflammatory cytokines, enhancing tumor neoantigen presentation, and potently increasing cytotoxic activity of tumor infiltrating lymphocytes. These studies are finding success in several pre-clinical models, and are entering early phases of clinical trial. Here, we review pre-clinical studies of RLR agonists, including the successes and challenges currently faced RLR agonists on the path to clinical translation.

## INTRODUCTION

The immune system is capable of targeted tumor cell killing through the process of immunosurveillance. Although tumors often develop ways to escape immunosurveillance, the growing interest and understanding of molecular interactions that occur between the tumor and the immune system have resulted in treatment strategies aimed at harnessing the immune system to target cancers. Recent advances in tumor immunology have produced immune checkpoint inhibitors (ICIs), cancer treatments designed to relieve the checkpoint restraints on adaptive immunity [1]. ICIs have revolutionized treatments for many types of cancer [1–3]. Despite these successes, not all patients respond to ICI therapy, for reasons that are varied and incompletely understood. It is thought that ICIs may be less effective in tumors that are poorly immunogenic, as defined by low levels of tumor infiltrating lymphocytes (TILs), minimal cross-presentation of tumor neoantigens, and high levels of immune suppressive leukocytes such as regulatory T-cells (T_Reg_s), tumor associated macrophages (TAMs) and myeloid derived suppressor cells (MDSCs) [4–7]. Innovative strategies to increase immunogenicity in tumors are being explored through a variety of approaches. One emerging strategy is based on activation of innate immunity in the tumor microenvironment (TME) [8, 9]. Innate immunity is a powerful arm of the immune system responsible for rapid anti-microbial immunity, often inducing programmed cell death of an infected cell. Innate immunity functions beyond the infected cell as well, by modulating the expression of cytokines and chemokines that recruit T-lymphocytes to the affected tissue, enhance antigen presentation, and increase cross-priming to antigen-specific T-cells [8, 10]. This idea is being explored extensively in regards to the pattern recognition receptor (PRR) known as Stimulator of Interferon Genes (STING) [11, 12]. Synthetic STING ligands potently induce anti-tumor immunity in several cancers, including breast cancer, chronic lymphocytic leukemia, colon cancer, and squamous cell carcinoma [13–17]. However, there is increasing evidence that STING signaling might be defective in some cancers, due to mutations, promoter methylation, and decreased expression of STING pathway effectors [18, 19], thus limiting their potential efficacy in the tumor cell compartment of the TME. However, other cells of the TME, particularly cells of the immune compartment, may retain STING signaling even when the STING pathway is defective within the tumor cells, per se, allowing STING ligands to induce innate immunity within the TME under these circumstances [20].

**Figure 1:**
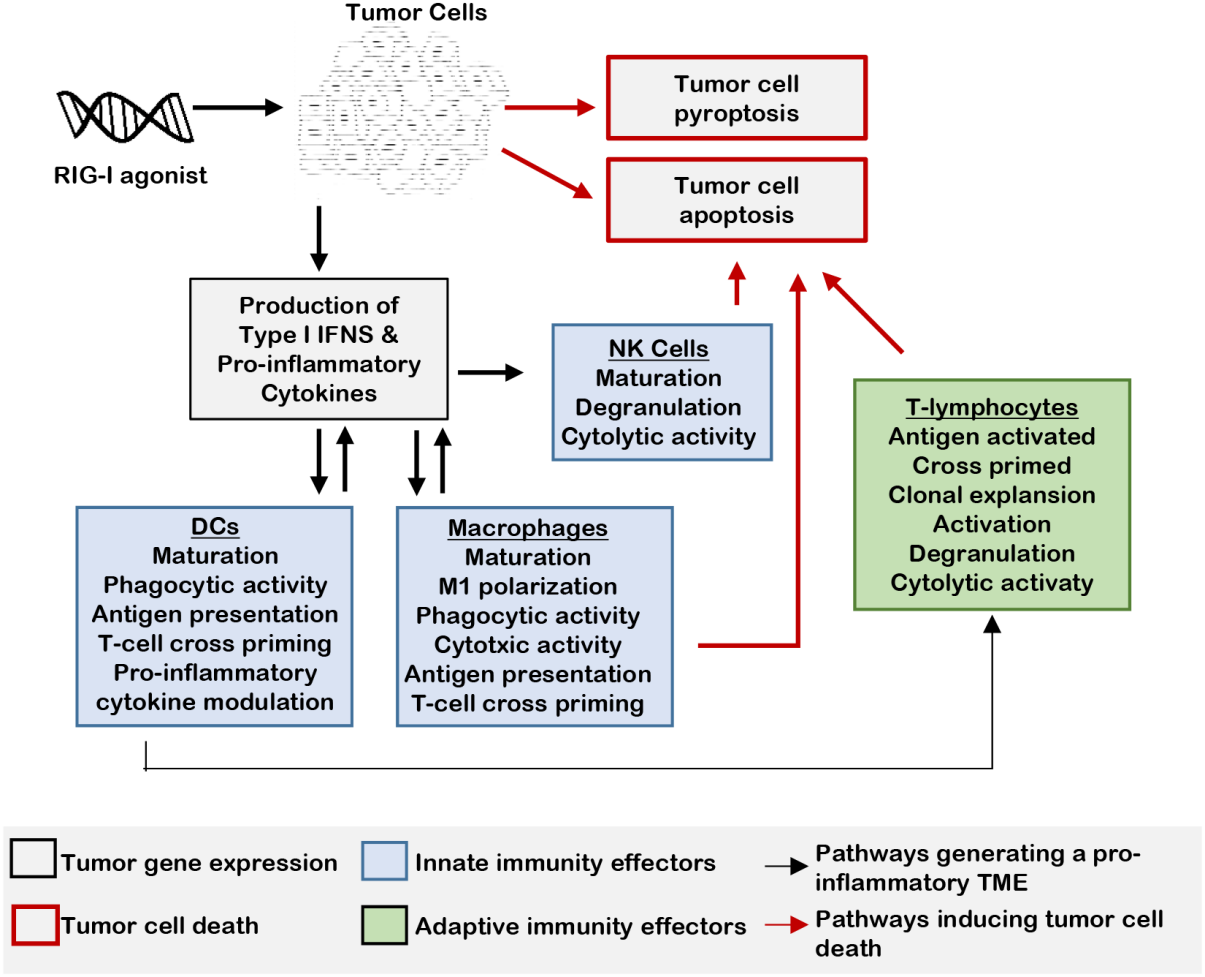
RLR activation signals innate immunity in the TME When tumor cells are treated with an RIG-I mimetic, inflammatory cytokine and type I IFN expression is rapidly upregulated, inducing innate immune responses in the tumor microenvironment. The cytolytic activity of leukocytes, such NK cells and macrophages, is increased in response to this IFN-enriched microenvironment. Maturation and activation of macrophages and DCs result in enhanced antigen presentation to T-lymphocytes in tumor draining lymph nodes. T-regulatory cell differentiation is decreased by the pro-inflammatory microenvironment produced by RIG-I activation.

Viral nucleic acid sensors, such as the RNA helicase known as retinoic acid-inducible gene I (RIG-I, encoded by the gene *DDX58*) [21], are expressed in most cells of the human body, including tumor cells [22]. When infected by an RNA virus, double-stranded RNA replication intermediates derived from the virus bind to RIG-I [23–26] and activate a RIG-I inflammasome leading to pyroptosis, a highly immunogenic mechanism of programmed cell death [27–29]. A hallmark of pyroptosis is the formation of pores in the plasma membrane [30], leading to hypotonic cell swelling and leakage of intracellular contents, including danger associated molecular patterns (DAMPs), into the microenvironment. RIG-I signaling simultaneously induces expression of pro-inflammatory cytokines [8, 10]. Together, DAMPs and pro-inflammatory cytokines stimulate a local acute inflammatory immune response aimed at removal of virus and virally-infected cells [31]. Interestingly, viral nucleotide motifs can be mimicked using synthetic, non-infectious oligonucleotides. These RIG-I agonists are capable of triggering RIG-I signaling, pyroptosis, and acute inflammation [26, 32–35]. In the cancer setting, RIG-I activation could thus provide a three-pronged attack: 1.) direct activation of tumor cell death; 2.) cytokine-mediated activation of innate immune effectors (e.g., macrophages, natural killer cells), and 3.) increased recruitment and cross priming of adaptive immune effectors (*e.g*., CD8+ T-lymphocytes) through a cytokine-enriched microenvironment and enhanced activity of professional antigen presenting cells [APCs, *e.g*., dendritic cells (DCs) or macrophages] (Figure 1). Synthetic RIG-I agonists are being explored as a therapeutic approach in a diverse range of cancers [27, 33, 34, 36]. Here, we review studies of RIG-I signaling in the tumor microenvironment, and preclinical studies investigating RIG-I agonists for cancer treatment.

### Activation of RIG-I induces pro-inflammatory signaling in a cell-intrinsic manner

RIG-I was first identified as a cytosolic DExD/H box RNA helicase activated in response to certain RNA viruses [21]. RIG-I is activated upon recognition of its ligand, double-stranded RNA sequences modified with a 5′-triphosphate (5′-3pRNA) or 5′-diphosphate (5′-2pRNA) motif [24, 26, 27, 37]. RIG-I activation may occur in response to other RNA motifs, including blunt dsRNAs [38], monomeric RNA within defective human immunodeficiency virus (HIV)-1 particles [39], cytoplasmic long non-coding RNAs [40], small nuclear RNAs [41–44], or endogenous retroviral transcripts. In addition to the DexD/H box RNA helicase domain, RIG-I is characterized by an amino-terminal Caspase Activation and Recruitment Domain (CARD) domain, and a Carboxy-Terminal Domain (CTD) [45–47]. Once activated by its ligand, RIG-I undergoes an ATP-dependent conformational change, exposing its CARD domain for polyubiquitylation [48] by ubiquitin ligases such as TRIM25, Riplet and others [49–52]. Once polyubiquitylated, a mitochondrial signalosome, comprised of the proteins WHIP, PPP6C and TRIM14, recruits RIG-I to the mitochondrial surface where the CARD domain of RIG-I interacts with the CARD domain of Mitochondrial Anti-Viral Signaling (MAVS), a requisite RIG-I co-factor [49, 53–55].

**Figure 2:**
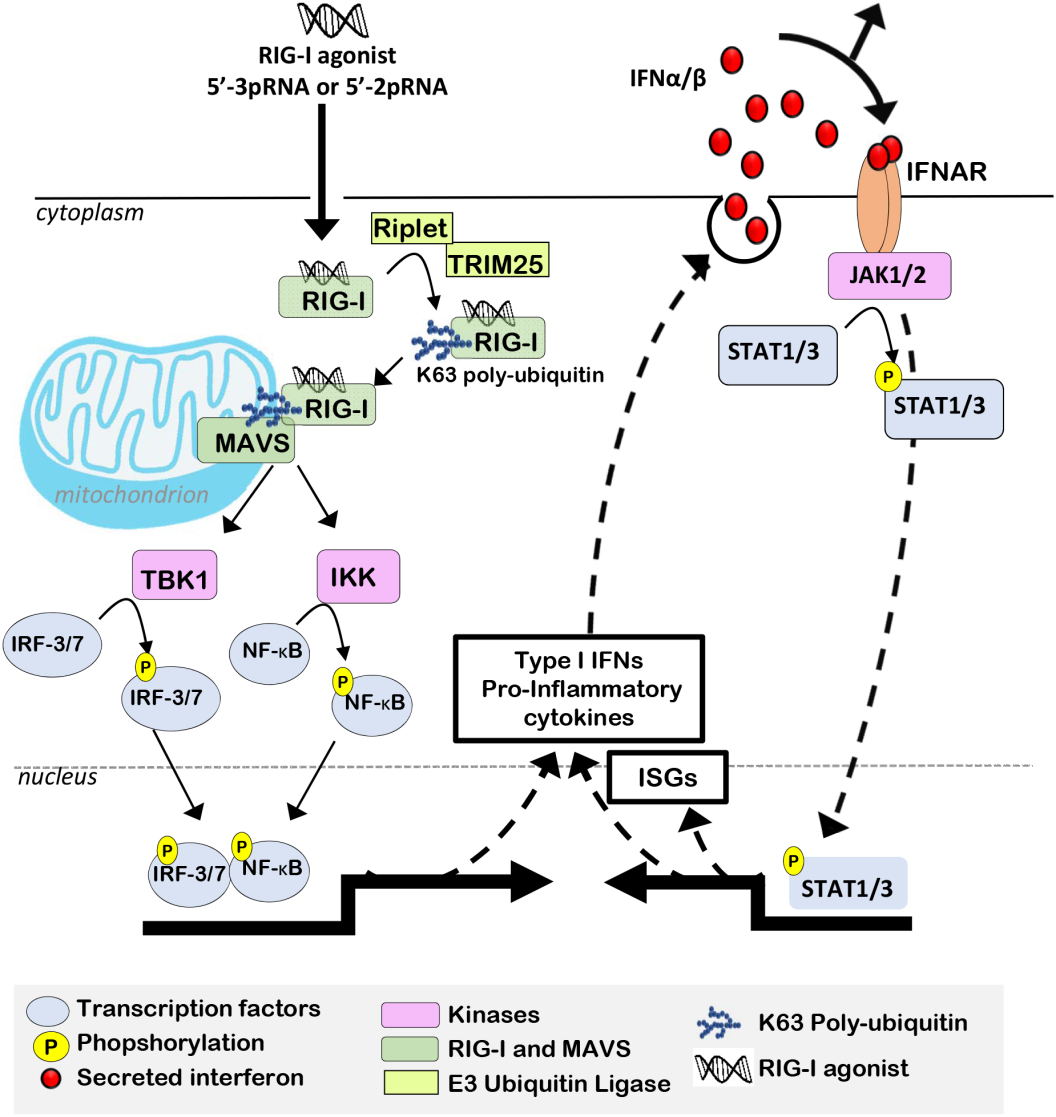
RIG-I activation induces Type I IFNs, which support pro-inflammatory transcriptional reprogramming RIG-I binding to 5′-3pRNA or 5′-2pRNA induces a conformational change, allowing RIG-I CARD domains to be polyubiquitylated by E3 ligases (*e.g*., Riplet or TRIM25). Polyubiquitylated RIG-I is recruited to mitochondria outer membranes, where it interacts with MAVS, which then activates IKK-ε, IKK-γ, and TBK1, kinases responsible for phosphorylation/activation of transcription factors (ATF-1, c-Jun, CBP, IRF-3, NF-κB). These transcription factors induce an expression profile that includes Type I I IFNs and additional pro-inflammatory cytokines. Type I IFNs bind to IFNAR, activating the intracellular tyrosine kinase JAK1/2, which in turn phosphorylates pro-inflammatory STAT transcription factors, thus driving expression of additional ISGs and amplifying the IFN-inducible positive feedback loop to support and maintain a pro-inflammatory microenvironment.

Once engaged, MAVS signaling activates three kinases that serve as regulators of inflammation, Inhibitor of κB-Kinase (IKK)-γ, TANK-Binding Kinase (TBK)-1 and IKK-ε [56–58]. These kinases phosphorylate Interferon (IFN) Regulatory Factor (IRF)-1, IRF-3, IRF-7, and Nuclear Factor (NF)-κB [59–61], transcription factors that drive expression of a pro-inflammatory transcriptional program that includes type I IFNs and pro-inflammatory cytokines [45, 62]. Importantly, IFN-α, IFN-β, and other pro-inflammatory cytokines produced in response to RIG-I activation drive a feed-forward signaling loop that maintains high expression levels of RIG-I, IFNs and additional pro-inflammatory IFN-stimulated genes (ISGs), by maintaining phosphorylation and activation of the transcription factors IRF-3, IRF-7, and NF-κB, and by phosphorylation of the transcription factor Signal Transducer and Activator of Transcription (STAT)-1, which occurs in response to IFN-α/β receptor (IFNAR)-mediated activation of JAK-STAT signaling (Figure 2) [62]. This feed-forward signaling model amplifies inflammatory cytokine production in the infected and neighboring cells, while recruiting leukocytes to the infected area, including pro-inflammatory lymphocytes. Since a ‘T-cell inflamed’ microenvironment is often associated with an improved prognosis for several cancers, and correlates with increased tumor sensitivity to ICIs, the pro-inflammatory phenotype induced by RIG-I activation may be an attractive treatment approach to increase tumor immunogenicity and clinical success of ICIs.

Two RIG-I-like receptors (RLRs) with structural similarity to RIG-I have been identified. One of these RLRs, Melanoma Differentiation Associated (MDA)-5, harbors an amino-terminal CARD domain, a DexD/H box motif, and a CTD domain [63, 64]. Like RIG-I, MDA-5 induces type I IFNs and other pro-inflammatory cytokines in response to viral nucleotides, albeit viral nucleotide motifs that are distinct from those that activate RIG-I. MDA-5 is activated by blunt-ended, long double-stranded RNA [*e.g.* polyinosinic-polycytidylic acid, or poly(I:C)], a ligand that also activated some Toll-Like Receptors (TLRs). In contrast to RIG-I and MDA-5, the other RLR known as Laboratory of Genetics and Physiology (LGP)-2 lacks the CARD domain shared by RIG-I and MDA-5, but is otherwise similar to the other RLRs [65]. Without the CARD domain, LGP-2 is unable to interact directly with MAVS to initiate a pro-inflammatory response. There are reports suggesting that LGP-2 activation interferes with RIG-I signaling, but that MDA-5 signaling may be enhanced by LGP2 [48, 66–69]. The implications of LGP2 expression and signaling in the context of cancer therapy, and how LGP2 might affect therapeutic responses to RIG-I agonists, are currently unclear.

### RIG-I signaling potently activates programmed cell death

In the context of viral infection, RIG-I signaling is capable of inducing programmed cell death (PCD) as a mechanism to eliminate virally-infected cells. Cellular mechanisms by which RIG-I induces PCD include activation of the intrinsic apoptosis pathway, the extrinsic apoptosis pathway, and a type of programmed necrosis termed ‘pyroptosis.’ The molecular factors governing the mode of RIG-I mediated cell death may depend to some extent on cell type. For example, RLR activation in keratinocytes, melanoma cells, glioblastoma cells, and many leukemia cells cause mitochondrial outer membrane permeabilization (MOMP), cytochrome-C release from mitochondria, and activation of caspase-9 and Apaf-1, the irreversible molecular switch that governs the intrinsic apoptotic pathway [27]. However, RIG-I signaling in pancreatic and prostate cancer cells robustly induces expression of several factors that activate the extrinsic apoptotic pathway, including Fas, Fas Ligand, Tumor Necrosis Factor (TNF), TNF-related apoptosis-inducing ligand (TRAIL), and the TRAIL receptors Death Receptor (DR)-4 and DR-5, causing caspase-8 activation and extrinsic apoptosis. The mechanism by which RIG-I signaling upregulates TRAIL, FAS and other extrinsic apoptosis-activating factors are not entirely clear, although it is likely that IFN signaling is involved, given that Fas, TRAIL, and caspase-8 are known ISGs [70, 71].

**Figure 3:**
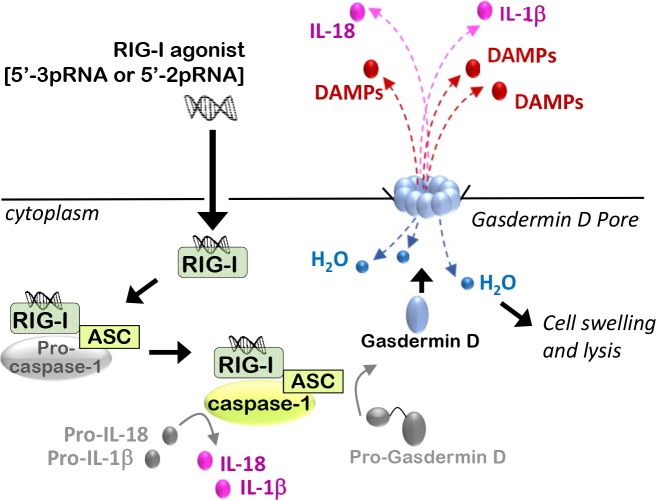
RIG-I activation induces immunogenic modes of programmed cell death Activated RIG-I recruits the inflammasome adaptor protein ASC, which facilitates binding and oligomerization of Caspase-1, leading to caspase-1 auto-cleavage and activation. Caspase-1 cleaves protein precursors of IL-1β and IL-18 to generate their mature, pro-inflammatory isoforms, which are then secreted. Caspase-1 activity also drives cleavage of the auto-inhibitory domain from Gasdermin-D, liberation the amino-terminal pore-forming domain of Gasdermin-D to translocate to the plasma membrane and oligomerize, forming pores that initiate hypotonic cellular swelling and lysis, followed by release of DAMPs into the extracellular space, thus inducing an inflammatory response from surrounding cells.

Another mode of programmed cell death induced upon RIG-I activation is termed “pyroptosis,” an immunogenic form of cell death occurring in response to activation of the inflammasome, a multi-protein holoenzyme comprised of capsase-1 oligomers, adaptor proteins known as ASC (Apoptosis-associated Speck with a Caspase-recruitment domain), and a molecular sensor of pathogens, such as RIG-I (Figure 3). RIG-I can interact, via its CARD domain, with the CARD domains of inflammasome components [72], resulting in auto-cleavage and activation of caspase-1 [29, 73], which then allows proteolysis of the pro-inflammatory cytokines interleukin (IL)-1β and IL-18 [73], which amplify inflammatory signaling in the local environment while activating natural killer (NK) cells and recruiting leukocytes to the affected tissue. Caspase-1 activation also results in cleavage of Gasdermin-D, removing the auto-inhibitory domain from Gasdermin-D to allow oligomerization at the plasma membrane and pore formation. Plasma membrane permeabilization by Gasdermin-D pores allows water to enter and swell the cell, a hallmark of necrosis. Once membrane integrity is lost, intracellular contents, including DAMPs, permeate the extracellular environment, inducing danger responses in neighboring cells, which amplifies the inflammatory response.

### RIG-I signaling in tumor cells affects the complex tumor microenvironment

The capacity for RIG-I signaling to induce cell death, while inducing pro-inflammatory responses, makes therapeutic use of RIG-I mimetics a highly attractive option in cancers. A growing number of studies show that the molecular responses to RIG-I or RLR signaling are retained in tumor cells and in non-tumor cells of the tumor microenvironment, and support innate immune responses against tumor cells [34]. For example, RIG-I activation in ovarian cancer cells enables NK-mediated tumor cell killing in culture [36]. Further, RIG-I signaling within the tumor cell increases phagocytosis of the affected tumor cell by professional APCs, including macrophages and DCs, thus providing tumor antigens for presentation to lymphocytes [32]. At the same time, the IFN-enriched microenvironment generated by tumor cell RIG-I signaling increases expression of major histocompatibility complex (MHC)-II antigen presentation molecules in macrophages and DCs, which may further increase tumor antigen cross-presentation. In support of this idea, it is reported that DCs presented pancreatic cancer-derived antigens more robustly to T-cells if RIG-I signaling was activated in pancreatic cancer cells prior to their co-culture with DCs [32, 36]. Similar results were observed upon RIG-I activation ovarian cancer cells prior to co-culture with macrophages [74].

### RIG-I mimetics are gaining traction as a possible cancer treatment in pre-clinical studies

Through direct activation of intrinsic immunity in cancer cells, and accompanying indirect activation of leukocytes in the TME, synthetic RIG-I mimetics are under investigation for cancer treatment in pre-clinical studies in hepatocellular carcinoma [75], leukemias [76], melanomas [27], prostate cancers [77] and others. RIG-I agonists that are stable and functional *in vivo* are under current development. For example, a minimal 5′-triphophosphorylated stem-loop RNA (SLR) sequence for intra-venous delivery to mice was recently reported [25]. The stem-loop structure enhances structural stability of the complex, a key determinant of RIG-I ligand potency. Delivery of SLR sequences to mice *in vivo* activated RIG-I signaling, IFN induction, and expression of genes required for potent anti-viral immunity, although this RIG-I mimetic has not yet been studied in tumors grown *in vivo*. A pre-clinical compound specific for RIG-I is RGT100 (Merck/Rigontec), currently in phase I clinical trials for treatment of advanced solid tumors and lymphomas (NCT03065023), although peer-reviewed preclinical reports for RGT100 were not identified, to our knowledge. Another compound which activates RIG-I by unknown mechanisms is SB-9200 [78], which is currently under investigation as an anti-viral agent, but has not yet been tested in the pre-clinical setting of cancer treatment.

In addition to RIG-I specific mimetics, synthetic RLR mimetics are being investigated in pre-clinical and early clinical studies. The compound Hiltonol [polyinosinic-polycytidylic acid stabilized with poly-l-lysine and carboxymethylcellulose (poly-ICLC)] [79, 80] was tested in combination with chemotherapy for patients with Stage IV anaplastic astrocytoma, resulting in increased overall survival (OS) to >8 years, versus the expected survival of two years on conventional chemotherapy alone [81]. Another trial tested poly-ICLC in combination with radiation and temozolomide in newly diagnosed adult glioblastoma patients. In these studies, intramuscular poly-ICLC increased OS to 18.3 months from 14.6 months [82–84]. Further, poly-ICLC is being tested as a tumor vaccine adjuvant in several cancer types, with a growing number of successes in Phase I and II clinical trials for gliomas [85], breast cancer [86], pancreatic cancer [87], ovarian cancer [88, 89], multiple myeloma [90], and others, highlighting the potential advances that Poly-ICLC may achieve across a spectrum of cancers. Although poly-ICLC potently activates MDA-5, it also activates Toll-like Receptor (TLR)-3, making the specific contributions of RLR signaling to the therapeutic effects of poly-ICLC, and to patient outcome, difficult to dissect.

### The future of RIG-I agonists in cancer

Exciting innovations within the field of RIG-I agonists are emerging. For example, a powerful, bimodal application of RNAi-based silencing of intra-tumoral gene targets using a 5′-triphosphate modified dsRNA sequence would allow for RIG-I activation and simultaneous gene targeting. This approach was demonstrated in melanomas, using 5′-3p-siRNA sequences specific to the anti-apoptotic gene *BCL2*. Delivery of this construct to cells potently stimulated IFN production and NK activation, while enhancing tumor cell killing through Bcl-2 ablation [34]. This concept was validated using a 5′-3p-siRNA targeting transforming growth factor (TGF)-β in pancreatic cancer cells, resulting in tumor cell apoptosis, IFN induction, and enhanced CD8+ T cell responses [36]. A similar approach was used in models of non-small cell lung cancer, using 5′-3p-siRNA sequences against vascular endothelial growth factor (VEGF), resulting in reduced tumor angiogenesis while enhancing anti-tumor immunity [91]. Defining the most appropriate gene silencing target may be a difficult task, but the use of siRNA paves the pathway for targeting certain oncogenes (e.g., *MYC*) that are currently ‘undruggable.’

Despite the potential success of RIG-I and RLR agonists, the immune system is powerful and incompletely understood, warranting cautious optimism and thorough examination of the caveats associated with innate immune activation, including possible on-target induction of autoimmunity, or induction of a cytokine ‘storm’ which could pose a threat to patient safety [92–94]. It is important to note that, since RIG-I is expressed in most cells of the human body, the consequences of RIG-I activation might be widespread, driving symptoms like fatigue, depression and cognitive impairment. In ICI-based therapies, these side-effects are generally managed by corticosteroid immunosuppression.

Delivery of small nucleotide sequences to tumor cells and leukocytes within the TME is another major obstacle to the widespread utility of RIG-I or RLR-based therapeutics in the cancer setting. Studies aimed at generating stable, specific and potent RIG-I ligands that retain functionality *in vivo* have been reported only recently. For example, a study employing a minimal 5′-triphophosphorylated stem-loop RNA (SLR) sequence delivered by intra-venous delivery to mice activated in RIG-I signaling, IFN induction, and expression of genes required for potent anti-viral immunity *in vivo*. A recently described ‘conditional’ RIG-I ligand, in which the 5′-triphosphorylated terminus of the RNA duplex remained shielded until release by predetermined molecular cues *in vivo*, could enhance delivery of RIG-I agonist to tumors, and minimize RIG-I activation outside of the TME [95]. However, the efficacy of RIG-I ligands, including SLRs and conditional RIG-I ligands, not yet been tested in animal models of cancer [25].

## CONCLUSION

Therapeutic RIG-I and RLR agonists are emerging as a novel approach to engage the immune system in the fight against cancer. Importantly, RIG-I signaling directly promotes tumor cell killing through three distinct modes of action: intrinsic apoptosis, extrinsic apoptosis, and pyroptosis. Further, simultaneous activation of the innate and adaptive arms of the immune system may generate durable therapeutic responses. The multi-faceted mechanisms by which RLR agonists eliminate cancer cells represent the well-rounded arsenal of weapons required to fight aggressive and metastatic cancers effectively.

## Abbreviations

APC: antigen presenting cell
ASC: Apoptosis-associated Speck with a Caspase-recruitment domain
CARD: caspase activation and recruitment domain
CTD: carboxy-terminal domain
DAMP: danger associated molecular patterns
DC: dendritic cell
DR: death receptor
HIV: human immunodeficiency virus
ICI: immune checkpoint inhibitors
IFN: interferon
IFNAR: interferon α/β receptor
IKK: inhibitor of κB-kinase
IL: interleukin
IRF: interferon response factor
ISG: interferon stimulated genes
MAVS: mitochondrial anti-viral signaling
MDSC: myeloid derived suppressor cell
MDA-5: melanoma differentiation antigen-5
MHC: major histocompatibility complex
MOMP: mitochondrial outer membrane permeabilization
NF: nuclear factor
NK: natural killer
OS: overall survival
PCD: programmed cell death
Poly(I:C): polyinosinic-polycytidylic acid
Poly-ICLC: polyinosinic-polycytidylic acid stabilized with poly-l-lysine and carboxymethylcellulose
PRR: pattern recognition receptor
RIG-I: retinoic acid-inducible gene I
RLR: RIG-I-like receptor
STAT: Signal transducer and transcription factor
STING: Stimulator of Interferon Genes
TAM: tumor associated macrophage
TBK: tank binding kinase
TGF: Transforming growth factor
TLR: toll-like receptor
TME: tumor microenvironment
TNF: tumor necrosis factor
TRAIL: TNF-related apoptosis-inducing ligand
T_Reg_: regulatory T-cells
VEGF: vascular endothelial growth factor
5′-3pRNA: 5′-triphosphate RNA
5′-2pRNA: 5′-diphosphate
